# Supplementary material for: Comparison of Citrated Whole Blood to Native Whole Blood for Coagulation Testing Using the Viscoelastic Coagulation Monitor (VCM Vet™) in Horses
Source: Animals (Basel). 2024 Oct 8;14(19):2892. doi: 10.3390/ani14192892 (PMC11476484; doi:10.3390/ani14192892)
Supplement: Supplementary file 1 [file animals-14-02892-s001.zip › animals-3233783-supplementary.pdf]

Table S1. Signalment for enrolled horses. BCS, body condition score; BW, body weight; NZ, New Zealand. Estimated body weights are indicated by *. Horses 1–20 represent horses meeting the inclusion criteria for determination of reference intervals.

Horse	Age (year)	Breed	Sex	BCS	BW (kg)	Reason for Presentation
1	18	Standardbred	Gelding	5.5	520	Routine dental
2	14	Standardbred	Gelding	4.5	602	Research
3	6	NZ Sporthorse	Mare	6	558	Lameness (forelimb CT)
4	9	Thoroughbred	Gelding	5	570	Routine dental
5	7	Friesian cross	Stallion	5	672	Routine surgery (castration)
6	7	Warmblood	Mare	5	570	Reproduction (pregnancy diagnosis)
7	6	Warmblood	Mare	7	614	Skin mass
8	13	Thoroughbred cross	Gelding	7	484	Eyelid mass
9	8	Warmblood	Stallion	7	680	Lameness work-up
10	3	Warmblood	Mare	4	440	Skin mass
11	9	Thoroughbred	Mare	5.5	630	Skin masses
12	7	Crossbred	Gelding	6	449	Lameness work-up
13	7	Gypsy cob cross	Gelding	5	296	Skin mass
14	5	Quarter Horse	Mare	6.5	483	Ophthalmic examination (corneal lesion)
15	4	Spanish Andalusian	Gelding	6	502	Ophthalmic examination
16	5	Thoroughbred	Gelding	3	470	Euthanasia (behavioral)
17	10	Quarter Horse cross	Mare	5	500 *	Skin mass
18	19	Standardbred	Gelding	6	652	Research
19	16	Standardbred	Gelding	5	566	Research
20	8	Arabian cross	Gelding	4.5	430	Routine dental
21	5	Appaloosa	Gelding	5.5	470	Lameness (chronic laminitis)
22	1.5	Thoroughbred	Colt	5.5	401	Discharging tract (mandible)
23	15	Arabian cross	Mare	4	435	Oral examination (foreign object)
24	1	Stationbred	Mare	5	393	Companion
25	6	Thoroughbred	Mare	4	500 *	Euthanasia (behavioral)
26	1	Quarter Horse	Colt	5	380	Routine surgery (castration)
27	21	Warmblood	Gelding	5.5	624	Research
28	17	Stationbred	Mare	5	545	Ophthalmic examination (chronic uveitis)
29	16	Thoroughbred	Gelding	4.5	562	Discharging tract (mandible)
30	18	Clydesdale cross	Mare	5	600 *	Colic (post-op small intestinal resection)
31	5	Crossbred	Mare	5.5	590	Behavioral complaints
32	10	Warmblood	Gelding	6	600 *	Respiratory signs
33	14	Warmblood	Mare	6	550	Respiratory signs (coughing)
34	6	Stationbred	Gelding	5	575 *	Lameness work-up
35	13	Warmblood	Mare	4.5	570	Companion
36	3	Clydesdale cross	Mare	7	650 *	Lameness work-up
37	4	Clydesdale cross	Mare	4	590	Eyelid mass
38	14	NZ Sporthorse	Gelding	4.5	475	Skin mass
39	12	Quarter Horse	Mare	6	568	Reproduction (breeding)
40	25	Thoroughbred	Mare	3	526	Weight loss
41	23	Standardbred	Mare	4.5	524	Lameness work-up
42	3	Stationbred	Colt	5.5	453	Routine surgery (castration)
43	13	Thoroughbred	Gelding	4	524	Lameness (septic navicular bursa)
44	14	Thoroughbred	Gelding	5	500 *	Lameness work-up
45	8	Standardbred	Gelding	5	502	Research

46	11	Kaimanawa	Mare	5	415	Behavioral complaints
47	6	Thoroughbred	Mare	4	534	Lameness (RF fetlock arthroscopy)
48	4	Gypsy cob cross	Mare	9	564	Reproduction (ultrasonography)
49	1.5	Thoroughbred	Filly	5	374	Routine surgery (epiglottic entrapment)
50	1	American miniature	Colt	5.5	80	Routine surgery (castration)
51	8	Thoroughbred	Gelding	5	632	Behavioral complaints
52	12	Spanish Andalusian	Gelding	5.5	564	Lameness work-up
53	6	Thoroughbred	Gelding	4	532	Euthanasia (chronic laminitis)
54	2	Quarter Horse cross	Colt	4.5	380	Routine surgery (castration)
55	15	Thoroughbred cross	Mare	4.5	530	Euthanasia (chronic lameness)
56	4	Stationbred cross	Gelding	5.5	480	Colic (large colon displacement)
57	14	Crossbred	Mare	5.5	400 *	Dental (dropping feed)
58	5	Stationbred cross	Stallion	4	494	Routine dental
59	7	Crossbred	Gelding	5.5	440	Routine dental
60	8	Thoroughbred	Gelding	5	564	Euthanasia (behavioral)
61	3	Friesian cross	Gelding	3	400 *	Enterocolitis
62	10	Crossbred	Gelding	5	510	Behavioral complaints
63	3	American miniature	Mare	7	108	Ophthalmic examination (chronic uveitis)
64	11	Warmblood	Gelding	5.5	540	Lameness work-up
65	10	Draught cross	Mare	7	680	Surgery (cystolith removal)
66	25	Warmblood	Mare	5	564	Lameness (chronic laminitis)
67	11	Warmblood cross	Gelding	4.5	522	Weight loss
68	6	Warmblood cross	Mare	3.5	600 *	Lameness work-up
69	11	Clydesdale cross	Gelding	5.5	460	Gastroscopy
70	23	Thoroughbred	Mare	4.5	522	Research
